# Comparison of efficacy between incretin-based therapies for type 2 diabetes mellitus

**DOI:** 10.1186/1741-7015-10-152

**Published:** 2012-11-30

**Authors:** Kaustubh Nisal, Ram Kela, Kamlesh Khunti, Melanie J Davies

**Affiliations:** 1 Department of Diabetes and Endocrinology, University Hospitals of Leicester NHS Trust, Leicester Royal infirmary, Infirmary Square, Leicester LE1 5WW, UK; 2 Department of Health Sciences, University of Leicester, 22-28 Princess Road West, Leicester LE1 6TP, UK; 3 Diabetes Department, Department of Cardiovascular Sciences, University of Leicester, Leicester General Hospital, Gwendolen Road, Leicester, LE5 4WP, UK

**Keywords:** GLP-1 analogues, GLP-1 agonists, DPP-4 inhibitors, incretins, head to head comparison, patient satisfaction

## Abstract

Type 2 diabetes mellitus is widely prevalent and is often coexistent with obesity. Many of the available treatment options have side effects such as weight gain which often affect patient's willingness to continue the treatment. Effective weight loss, lack of significant hypoglycaemia, and favourable cardiometabolic profile make Incretin based therapies an attractive treatment option for type 2 diabetes. Incretin based therapies are available as either incretin mimetics (also called GLP-1 agonists) or incretin enhancers (DPP-4 inhibitors). Although agents in both these classes of incretin based therapy are effective through a common GLP-1 pathway, there are many differences amongst them including the route of administration, frequency of administration, effects on body weight, extent of glycaemic improvement. There are several trials evaluating these individual incretin based agents either as monotherapy or in combination with other anti-diabetic agents, however very few have looked into direct comparison amongst the agents in these two classes. This review is aimed to look at important mechanistic differences between incretin mimetics and enhancers through direct comparison trials and impact of these differences on biochemical, metabolic and patient satisfaction parameters.

## Review

### Introduction

The prevalence of type-2 diabetes mellitus (T2DM) is rapidly increasing worldwide. The International Diabetes Federation (IDF) estimates the current prevalence of diabetes at around 366 million which is estimated to increase to 552 million cases of diabetes and 398 million cases of impaired glucose tolerance (IGT) by 2030 [1]. Similarly, there has been an uptrend in adiposity worldwide [2]. The National Obesity Observatory data estimates the prevalence of obesity in the UK at 23%, while 61% of adults are overweight [3]. The majority of patients with T2DM are obese [4] and many of the current therapeutic options for management of T2DM can cause further weight gain [5,6]. Concerns about weight gain adversely affect patients' willingness to begin and continue treatment with glucose-lowering medications, such as thiazolidinediones (TZDs), insulin, and sulfonylureas (SU) [7]. Often the patient's quality of life can be negatively affected by the underlying disease process and its complications, such as polypharmacy, weight gain, hypoglycemia and micro- and macro-vascular complications [8]. Recently introduced incretin based therapies appear to offer advantages over conventional therapies by either keeping the weight steady or even achieving weight loss and limiting hypoglycemia, while achieving effective glycemic control. This review examines the comparisons between two classes of incretin based therapies, dipeptidyl peptidase 4 (DPP-4) inhibitors (incretin enhancers) and glucagon-like peptide 1 (GLP-1) agonists (incretin mimetics). Although use of incretin based therapies for T2DM has been reviewed before [9-11], this article focuses on data from head to head comparative trials analyzing efficacy, tolerability and safety profiles between the agents from these two classes.

### Physiology of incretins

The classic 'incretin effect' refers to the observation that oral glucose elicits a higher insulin response compared to intravenous glucose at similar plasma glucose concentrations. GLP-1 and glucose-dependent insulinotropic polypeptide (GIP), two major incretin hormones that are secreted into the circulation by 'L' and 'K' cells of the small intestine, respectively, are responsible for 50% to 70% of glucose dependent insulin release [12-14]. Apart from insulinotropic effects, GLP-1 also suppresses glucagon release, reduces hepatic gluconeogenesis, delays gastric emptying and reduces food intake by promoting satiety [15]. The impaired incretin effect in patients with T2DM is thought to be multifactorial. Reduced postprandial GLP-1 response [16,17] and a reduced insulinotropic response [18] are contributing factors. In a study comparing healthy subjects with patients with T2DM, lack of the incretin effect, in spite of comparable GLP-1 as well as GIP secretion, was observed [19]. Administration of GLP-1 subcutaneously over six weeks in patients with T2DM has been shown to improve glycemic control, insulin sensitivity, and beta cell function along with reduced gastric emptying and reduction in bodyweight [15]. However, GLP-1 secretion is not always reduced and may be normal in patients with T2DM [20,21]. Age, body weight, fasting glucagon and Non-Esterified Fatty Acids (NEFA) concentrations are some of the factors thought to affect the incretin response [21].

### Incretin based therapies

Due to various favorable cardiometabolic and insulinotropic effects, GLP-1 is a very attractive candidate as a therapeutic invention in management of T2DM. Native GLP-1 has a very short half-life of a few minutes as it is broken down by endopeptidase enzymes such as DPP-4 which has a ubiquitous presence in the human body [22-24]. As the native GLP-1 molecule is unsuitable for routine clinical use, stimulation of GLP-1 receptors either by administration of GLP-1 agonists or restoring the endogenous GLP-1 pool by inhibiting its DPP-4 mediated breakdown are the two approaches used to obtain or maintain high levels of GLP-1[14].

### Incretin mimetics

GLP-1 agonists mimicking endogenous GLP-1 in their pharmacokinetic and pharmacological properties are termed incretin mimetics. These are modified GLP-1 molecules and are resistant to DPP-4 induced degradation. Exenatide, a molecule originally isolated from the saliva of the *Heloderma suspectum *lizard (Exendin-4) was the first GLP-1 agonist to become available for clinical use and was approved by the US Food and Drug Administration (FDA) in April 2005 and by the European Medicine Agency (EMA) in November 2006 [25,26].

Liraglutide is the first human GLP-1 analogue with 97% amino acids sequence homology with native GLP-1; fatty chain addition to its molecule prolongs its half-life to 13 hours [27]. Recently, a long acting once weekly preparation of exenatide (Bydureon) at a dose of 2 mg has been approved for clinical use by the EMA in Europe [28].

### Incretin enhancers

DPP-4 inhibitors are termed incretin enhancers as they prolong the half-life and availability of endogenous GLP-1 by inhibiting DPP-4. Sitagliptin was the first DPP-4 inhibitor approved for clinical use in October 2006 followed by vildagliptin in Europe and saxagliptin in the US markets [29]. Alogliptin has market approval in Japan, while linagliptin has recently gained approval for clinical use in the US as well as Europe.

Currently, other GLP-1 agonists (for example, lixisenatide and albiglutide) and DPP-4 inhibitors are at various stages of development and in clinical trials programs. Taspoglutide is another once weekly human GLP-1 analogue in development but further trials have been suspended in the late stages due to agent specific hypersentivity reactions.

### Comparisons between incretin mimetics (GLP-1 agonists) and incretin enhancers (DPP-4 inhibitors)

Agents in both these classes have been studied as monotherapy or in combination with other anti-diabetic medications. DPP-4 inhibitors are administered orally, once a day as compared to GLP-1 agonists which are administered subcutaneously, once or twice a day or more recently once a week. GLP-1 agonists slow gastric emptying in addition to a reduction in appetite but DPP-4 inhibitors do not seem to have these effects [30]. In general, the observation is that GLP-1 agonists have been found to be more effective in glycemic management and weight reduction compared to DPP-4 inhibitors. However, there are a limited number of head to head studies directly comparing the effects of DPP-4 inhibitors and GLP-1 agonists. The first data suggesting key differences between DPP-4 inhibitors and GLP-1 agonists comes from an initial short term proof of concept study suggesting important mechanistic differences between exenatide twice a day (bid) and sitagliptin [31]. Since then, longer term randomized control trials (RCTs) have compared these two classes of therapeutic agents as summarized in Table 1.

**Table 1:** Overview of head to head comparisons amongst GLP-1 analogues and DPP-4 inhibitors.

Study	Duration	Type	GLP-1 analogue	DPP-4 inhibitor	Co- existing therapy
DeFronzo *et al. *[31]	4 weeks	Double blind, double dummy, cross over	Exenatide 5 mcg twice daily for 1^st ^week followed by 10 mcg twice daily for 2^nd ^week	Sitagliptin 100 mg once daily	Metformin
Berg *et al. *[32]	8 weeks	Double blind, double dummy, cross over	Exenatide 10 mcg twice daily	Sitagliptin 100 mg once daily	none
1860-Lira DPP4 [33]	26 weeks	Open label parallel group	Liraglutide 1.2 mg and Liraglutide 1.8 mg	Sitagliptin 100 mg once daily	Metformin
DURATION 2 [35]	26 weeks	Double dummy	Exenatide QW 2 mg once weekly	Sitagliptin 100 mg once daily	Metformin
DURATION 4 [37]	26 weeks	Double dummy	Exenatide QW 2 mg once weekly	Sitagliptin 100 mg once daily	none
T-emerge 4 [39]	24 weeks	Double dummy	Taspoglutide 10 mg and 20 mg weekly	Sitagliptin 100 mg once daily	Metformin
1860- Lira DPP4 extension [34]	52 weeks	Open label parallel group	Exenatide 10 mcg twice daily	Sitagliptin 100 mg once daily	Metformin

DDP-4, dipeptidyl peptidase 4; GLP-1, glucagon-like peptide 1

### The 'proof of concept' study

In a short double blind, double dummy, cross-over study involving 61 patients with metformin treated T2DM, a two-week therapy with exenatide (5 mcg bid for the first week, increasing to 10 mcg bid for the second week) was associated with greater improvement in two-hour postprandial glucose (PPG) as compared to that obtained with two weeks of therapy with sitagliptin 100 mg once daily [31]. More importantly, sitagliptin-treated patients noticed further improvement in PPG levels after changing over to exenatide suggesting the superiority of exenatide in improving postprandial hyperglycemia, an effect of increased postprandial insulin release associated with GLP-1 receptor agonists. There was no statistically significant difference in the improvement achieved by both agents in fasting plasma glucose (FPG). The differential mechanistic effects are summarized in Table 2.

**Table 2:** Mechanistic differences between GLP-1 agonist exenatide and DPP-4 inhibitor sitagliptin [31].

		Sitagliptin	Exenatide	Significance
Change in FPG (mmol/l)		1.04+/- 0.2	0.83+/-0.2	*P *= 0.3234
Change in PPG (mmol/l)		2.0 +/-0.3	6.26+/-0.3	*P *<0.0001*
Insulinogenic index		Yes	Yes- significantly more than sitagliptin	*P *= 0.0239*
Acute Insulin secretion		Yes	Yes- significantly more than sitagliptin	*P *= 0.0017*
Reduction in post -prandial glucagon		Yes	Yes- significantly more than sitagliptin	*P *= 0.0011*
Reduction in gastric emptying		none	Yes- significantly more than sitagliptin	*P *<0.0001*
Six point SMBG excursions	Post breakfast		Yes- significantly less than sitagliptin	*P *= 0.0016*
	Post lunch		Similar to Sitagliptin	*P *= 0.07849
	Post dinner		Yes- significantly less than sitagliptin	*P *= 0.038*
Reduction in body weight (kg)		0.3+/-0.2	0.8+/-0.2	*P *= 0.0056*
Decrement in calorie intake		none	Yes- significantly more than sitagliptin	*P *= 0.0227*
Reduction in post-prandial triglyceride levels		yes	Yes- significantly more than sitagliptin	*P *= 0.018*
Nausea		12%	34%	
Vomiting		3%	24%	

*Statistically significant. DDP-4, dipeptidyl peptidase 4; FPG, fasting plasma glucose; GLP-1, glucagon-like peptide 1; PPG, postprandial glucose; SMBG, self-monitoring blood glucose

Patients' gastric emptying rates were also assessed using 1,000 mg of an oral liquid acetaminophen preparation. Exenatide significantly slowed gastric emptying compared to sitagliptin (*P *=<0.0001). Exenatide-treated patients were also found to exhibit a reduction in their calorie intake as assessed by *ad libitum *meals. There was reduced calorie intake averaging 134 kcal less in the exenatide-treated group compared to the sitagliptin-treated group. Due to the variability of the calorie intake, median caloric intake was assessed which showed a similar trend (exenatide: -138 kcal versus sitagliptin: +63 kcal).

During this two-week trial the mean postprandial glucagon concentration relative to baseline was reduced in both treatment groups; the reduction in postprandial glucagon following exenatide was significantly greater compared to sitagliptin (*P *=<0.0011). There was an increase in the insulinogenic index of insulin secretion with exenatide compared to sitagliptin (ratio exenatide to sitagliptin: 1.50 +/- 0.26, *P *= 0.0239). Nausea was the predominant side effect, experienced by 34% of patients treated with exenatide and 12% of patients treated with sitagliptin. Vomiting was experienced by 24% of patients treated with exenatide and 3% of patients treated with sitagliptin [31].

A more recent study comparing both of the above therapies given for eight weeks in patients with T2DM (baseline hemoglobin A1c (HbA1c) of 8.3 ± 1.0% and body mass index of 35 ± 5 kg/m2) revealed a reduction in postprandial glucagon secretion and an improvement in a homeostasis model assessment of beta-cell function (HOMA-B) with exenatide 10 mcg bid as well as sitagliptin 100 mg daily; however, the improvement was significantly more in exenatide treated patients compared to the sitagliptin treated group [32]. Both exenatide and sitagliptin therapies resulted in an improvement in two-hour PPG, average 24-hour glucose and the time spent with glucose between 3.9 and 7.8 mmol/L over a 24-hour period. However, exenatide therapy was associated with significantly lower two-hour PPG, average 24-hour glucose and more time spent with glucose between 3.9 and 7.8 mmol/l (*P *=<0.05). As recently observed in other studies, postprandial intact GLP-1 levels were reduced with exenatide therapy and increased with sitagliptin. Postprandial glucogon levels were reduced significantly more by exenatide therapy than sitagliptin (*P *=<0.005) [32].

To summarize, there appear to be important mechanistic differences between exenatide and sitagliptin in these short term studies. Longer term direct head to head comparative studies are needed to ascertain the durability and effects of these differences on the glycemic outcomes. Also, it is important to ascertain if these differential effects extend to the other agents in the respective incretin based classes.

### Head to head RCTs of GLP-1 agonists and DPP-4 inhibitors

The effect of these physiological differences were studied in four further randomized studies, each lasting for 24 to 26 weeks with one of them having a further extension period of 26 weeks (Table 1).

The 1860-Lira-DPP-4 study was an open label parallel group trial comparing liraglutide (1.8 mg and 1.2 mg) against sitagliptin (100 mg), all in combination with metformin in patients treated with T2DM [33]. Recently, the outcomes of an open label extension for a further 26 weeks in patients completing the 1860-Lira-DPP-4 study have been published [34]. Therefore, the 1860-Lira-DPP-4 study comparing liraglutide 1.2 and 1.8 mg with sitagliptin 100 mg is the longest head to head comparative study between a GLP-1 agonist and DPP-4 inhibitor.

The DURATION 2 (Diabetes Therapy Utilization: Researching changes in A1c, weight and other factors Through Intervention with exenatide ONce weekly) and DURATION 4 trials involved comparison of a recently approved once weekly preparation of exenatide (Exenatide QW 2 mg) against sitagliptin (100 mg/day) [35-37]. The DURATION 4 was a monotherapy study while the DURATION 2 involved combination therapy with metformin and also had a third arm involving pioglitazone [37]. In the T-emerge 4-trial, taspoglutide, a once weekly GLP-1 analogue, was compared against sitagliptin in a double-dummy 24 week trial [38]. Taspoglutide was suspended in the late stages of development due to concerns regarding hypersensitivity reactions and gastrointestinal side effects [39].

### Changes in HbA1c

In the 1860-Lira-DPP-4 study, mean baseline HbA1c was 8.4%. A greater reduction in HbA1c was seen with liraglutide 1.2 mg (−1.24%; 95% CI, −1.37 to −1.11) and 1.8 mg (−1.5%; 95% CI, −1.63 to −1.37) compared with sitagliptin (−0.9%; 95% CI, −1.03 to −0.77). Estimated mean treatment differences for liraglutide at 1.2 mg and 1.8 mg doses compared to 100 mg sitagliptin were −0.34% for 1.2 mg (*P *<0.0001) and −0.6% for 1.8 mg (*P *=<0.0001). The reduction of 0.9% in HbA1c with sitagliptin in the 1860-Lira-DPP-4 study is one of the better results achieved in a trial with sitagliptin [33].

During the extension phase of the 1860-Lira-DPP-4 study, mean HbA1c improvement with liraglutide 1.8 mg and 1.2 mg, and sitagliptin at 52 weeks from baseline was 1.29%, 1.51% and 0.88%, respectively. Thus liraglutide produced a significant and sustained reduction in HbA1c compared to sitagliptin at 52 weeks. The improvement in glycemic control with liraglutide was irrespective of baseline HbA1c [34].

In the DURATION-2 study, the mean baseline HbA1c was 8.6%. Exenatide QW therapy resulted in a significant reduction in HbA1c compared with sitagliptin (−1.5% versus −0.9%, *P *=<0.0001). Significant HbA1c improvement was noted within four weeks of exenatide QW therapy and within six weeks of sitagliptin therapy. In a subgroup of patients with a basal HbA1c less than 9%, exenatide QW therapy resulted in significant improvements (mean baseline HbA1c 7.8%, change in HbA1c -1.1%) in comparison to sitagliptin (mean baseline HbA1c 7.7%, change in HbA1c -0.5%) [35]. It is well appreciated that the relative contribution of PPG in overall diurnal hyperglycemia is higher in well controlled subjects with diabetes [40]. Further improvement in HbA1c in a subgroup of well controlled patients during the DURATION-2 study therefore suggests underlying improvements in PPG, although PPG was not measured in the study.

In the T-emerge 4 Trial, taspoglutide 10 mg and 20 mg has been shown to improve HbA1c significantly more than that achieved with sitagliptin (-1.3%, -1.23% and -0.89% improvement from baseline with taspoglutide 20 mg, 10 mg and sitagliptin, respectively; *P *<0.001 for both doses of taspoglutide against sitagliptin). The mean baseline HbA1c across the treatment arms ranged from 7.95% to 8.03% in this study [38].

In the DURATION-4 trial 26 weeks monotherapy with exenatide QW reduced HbA1c by 1.5% from baseline as opposed to a 1.2% reduction with sitagliptin [37].

### Changes in glucose levels

In the 1860-Lira-DPP-4 study, the mean reduction in FPG was greater with liraglutide compared to sitagliptin (mean of -2.14 mmol/L with liraglutide 1.8 mg, -1.87 mmo/L with liraglutide 1.2 mg and -0.83 mmol/L with sitagliptin 100 mg) [33]. Improvements and differences in FPG were sustained during the extension phase of the 1860-Lira-DPP-4 study. At 52 weeks, the mean reduction in FPG was -2.04 mmol/l, -1.71 mmol/l and -0.59 mmol/l with liraglutide 1.8 mg, 1.2 mg and sitagliptin 100 mg, respectively [34]. Treatment differences between sitagliptin and liraglutide remained statistically significant for both doses (*P *<0.0001). The improvement in mean FPG was twofold greater with exenatide QW treated patients in comparison to sitagliptin treated patients in the DURATION-2 trial (-1.8 mmol/L versus -0.9 mmol/L, respectively) [35]. Changes in PPG levels were not assessed in these head to head trials. In contrast to short term mechanistic studies, there was a significant difference in FPG in these head to head comparative trials conducted over a longer period of time. Differences in efficacy and tolerability among studied GLP-1 analogue and DPP-4 inhibitor in the 1860-Lira-DPP-4 and DURATION-2 study are summarized in Table 3. In the DURATION 4 trial exenatide QW significantly reduced fasting glucose at 16 and 26 weeks as well 7 point self-monitoring blood glucose (SMBG) profiles compared to sitagliptin [37].

**Table 3:** Comparison of GLP-1 analogues in DPP-4 inhibitors- data from fully published RCTs [33-35,37].

Study	The 1860- Lira DPP-4 study (52 weeks)			DURATION 2 (26 weeks) - Add on therapy to Metformin		DURATION 4 (26 weeks) - Monotherapy	
**Agent**	**Liraglutide .8 mg/day**	**Liraglutide1.2 mg/day**	**Sitagliptin100 mg/day**	**Exenatide QW2 mg/weekly**	**Sitagliptin100 mg/day**	**Exenatide QW2 mg/weekly**	**Sitagliptin100 mg/day**

Number of patients	225	221	219	160	166	248	163
Mean baseline HbA1c (%)	8.4 (0.8)	8.4 (0.7)	8.5 (0.7)	8.6 (1.2)	8.5 (1.2)	8.4-8.6	8.4-8.6
Change in HbA1c (%)	-1.51	-1.29	-0.88	-1.5	-0.9	-1.53	-1.15 (*P *=<0.001)
Mean treatment difference in HbA1c with DPP-4 inhibitor	-0.63 (*P *<0.0001)	-0.4 (*P *<0.0001)	-	-0.6 (*P *<0.0001)	-	-	-
Mean baseline FPG (mmol/L)	10.1 (2.4)	9.9(2.4)	10.0 (2.0)	9.2(2.9)	9.1(2.5)	9.7 to 9.9	9.7 to 9.9
Change in FPG (mmol/L)	-2.04	-1.71	-0.59	-1.8	-0.9	-2.3	-1.1 (*P *=<0.001)
Mean treatment difference in FPG with DPP-4 inhibitor	-1.45 (*P *<0.0001)	-1.13 (*P *<0.0001)	-	-0.9 (*P *<0.0001)	-	-	-
Baseline weight (Kg)	93.7(18.4)	94.6(18.1)	93.1(18.9)	89(20)	87(20)	85.9 to 88.6	85.9 to 88.6
Change in weight (Kg)	-3.68	-2.78	-1.16	-2.3	-0.8	-2.0	-0.8 (*P *<0.001)
Mean treatment differences in weight with DPP-4 inhibitor	-2.53 (*P *<0.0001)	-1.62 (*P *<0.0001)	-	-1.5 (*P *<0.0001)	-	-	-
Incidence of hypoglycemia	0.143episodes/ patient/year	0.154 episodes/ patient/year	0.137 episodes/ patient/year	1%	3%	5.2%	3.1%
Nausea Number (%)	60 (27.5)	40 (21.7)	12 (5.5)	38 (24)	16 (10)	11.3%	3.7%
Diarrhea Number (%)	27 (12.4)	20 (9)	14 (6.4)	29 (18)	16 (10)	10.9%	5.5%

DDP-4, dipeptidyl peptidase 4; FPG, fasting plasma glucose; GLP-1, glucagon-like peptide 1; HbA1c, hemoglobulin A1c

### Changes in body weight

In the 1860-Lira-DPP-4 study group trial, the mean weight loss was significantly greater with liraglutide than sitagliptin. The estimated mean weight differences were -2.4 kg (95% CI-3.14 to -1.70) for 1.8 mg liraglutide versus sitagliptin, and -1.90 kg (-2.61 to -1.18) for 1.2 mg liraglutide versus sitagliptin. Liraglutide at both doses produced a greater reduction in waist circumference but there were no differences in waist to hip ratio [33]. During the 1860-Lira DPP4 extension phase, weight loss achieved during the first 26 weeks was sustained at 52 weeks. At the end of the study period mean weight loss with liraglutide 1.8 mg, 1.2 mg and sitagliptin was 3.68 kg, 2.78 kg and 1.16 kg, respectively with mean treatment differences between the agents remaining statistically significant (*P *<0.0001) [34].

In the DURATION 2 trial, the differences in weight loss became apparent by 4 weeks and by week 26, weight loss with exenatide QW (-2.3 kg, 95% CI -2.9 to -1.7) was significantly greater compared to sitagliptin (-0.8 kg, 95% CI -1.4 to -0.1). The mean treatment difference was -1.5 kg (95% CI -2.4 to -0.7, adjusted *P *= 0.0002) for exenatide QW versus sitagliptin. In terms of absolute numbers, more than 75% (n = 123) of patients on once weekly exenatide lost bodyweight compared with 61% (n = 101) of those on sitagliptin [35]. Weight loss with taspoglutide 10 mg and 20 mg once weekly dose was 1.23 kg and 2.54 kg, respectively, in comparison to 0.55 kg weight loss seen with sitagliptin over the 24 week study period [38]. In the DURATION 4 trial, treatment with exenatide QW reduced body weight significantly compared to sitagliptin (weight loss 2 kg versus 0.8 kg, *P *=<0.001) [37].

The effect of differential calorie intake and the reduced gastric emptying noticed during short term mechanistic studies between agents in the GLP-1 analogue and DPP-4 inhibitors groups probably explain the differential weight loss in favor of GLP-1 agonists in the subsequent longer term head to head comparisons up to a one-year period.

### Changes in blood pressure and other metabolic parameters

There was no significant difference observed for systolic blood pressure in the 1860-Lira-DPP-4 study group trial although both liraglutide and sitagliptin reduced the systolic blood pressure. Sitagliptin reduced diastolic blood pressure significantly compared to 1.8 mg liraglutide but there was no significant difference compared to 1.2 mg liraglutide. The overall effect on the blood pressure with either drug was small [33]. During the 1860-Lira-DPP-4 study extension there were no significant differences noted with liraglitide or sitaglipin except reduction of systolic blood pressure with 1.8 mg liraglutide. Other large clinical studies with liraglutide have shown consistent reductions in systolic blood pressure [41-46]. During the DURATION-2 trial the exenatide QW treated group had significantly lower systolic blood pressure at 26 weeks compared to sitagliptin. The mean difference was -4 mm Hg (CI -6 to -1 mm of Hg) between once a week exenatide and daily sitagliptin. There were no significant differences in the levels of diastolic blood pressure [35]. Similar to liraglutide, large clinical trials with exenatide have shown favorable effects on blood pressure [47]. DPP-4 inhibitors, on the other hand, have shown variable effects on blood pressure [48-50].

The 1860-Lira-DPP-4 study did not observe any significant differences with lipid profile except a significant reduction in total cholesterol from baseline with the 1.8 mg liraglutide dose compared to sitagliptin. In the DURATION 2 trial neither exenatide nor sitagliptin had any significant effect on the lipid profile.

### Hypoglycemia

In the DURATION-2 trial there were no reported major hypoglycemic episodes. Minor hypoglycemia episodes were similar with the exenatide QW and sitagliptin [35]. The 1860-Lira-DPP-4 study reported a single episode of major hypoglycemia with 1.2 mg liraglutide (blood glucose concentration of 3.6 mmol/L). Minor hypoglycemia episodes were reported by similar proportions of participants treated with 1.8 mg liraglutide (11 (5%), 0.370 episodes per participant-year), 1.2 mg liraglutide (12 (5%), 0.178), and sitagliptin (10 (5%), 0.106) [33]. During the extension phase of the 1860-Lira DPP4 study, no episodes of major hypoglycemia occurred and the minor hypoglycemia events remained comparable during the whole 52-week study period [34]. The DURATION 4 trial did not report any major hypoglycemia episodes. An incidence of 5.2% in the exenatide QW group versus 3.1% in the sitagliptin group was reported for unconfirmed hypoglycemia [51].

### Gastrointestinal side effects

As noticed in short term mechanistic studies, all the longer term comparative RCTs showed more initial nausea and vomiting with GLP-1 agonists compared to DPP-4 inhibitors. In the 1860-Lira-DPP-4 study, nausea was more common with liraglutide (21% to 27%) than with sitagliptin (5%) at the beginning of the therapy but by the end of the trial, symptoms decreased to the level observed with sitagliptin (<3%) and patients reported that nausea remained comparable during the extension period [33,34]. In the DURATION-2 trial nausea was more common with once a week exenatide (24% patients) compared to sitagliptin (10% patients) [36]. The DURATION 4 trial reported 11.3% patients experiencing nausea on treatment with exenatide QW while vomiting was noted in 4.8% patients treated with exenatide QW compared to 1.8% patients in the siatgliptin group [51].

### Incretins and safety

Cases of pancreatitis have been reported in the patients who were treated with agents in both classes of incretin-based therapies [52]. During the head to head comparison trials, no episode of pancreatitis was noticed during the first 26 weeks of the 1860-Lira-DPP4 study. However, an episode of mild non acute pancreatitis was reported during the extension period [34]. No cases of pancreatitis were reported during the DURATION-2 trial.

Large preclinical studies involving diabetic mice and rats have failed to show an association between GLP-1 agonists, such as exenatide and liraglutide, as well as the DPP-4 inhibitor sitagliptin and pancreatitis [53,54]. Large cohort studies looking at the health care databases have not shown any association with the incretin-based therapies and pancreatitis [55,56]. A recently published large cohort study analyzed the rates of acute pancreatitis in diabetic subjects treated with exenatide, sitagliptin and other antidiabetic agents using data from the Medco National Integrated Database from January 2007 to June 2009. The risk of pancreatitis was high in patients with diabetes compared to patients without diabetes (adjusted hazard ratio 2.1 (95% CI 1.7 to 2.5)), but there was no increased risk of pancreatitis seen in patients treated with exenatide or sitagliptin compared to patients who received other diabetic medications [57]. The available data do not support an association between incretin therapies and pancreatitis. Long term larger studies are needed to investigate this further.

Long term exposure to liraglutide has been shown to be associated with thyroid 'C' cell hyperplasia in rodents [58]. In contrast, monkeys and humans have much lower levels of GLP-1R expression, and prolonged administration of liraglutide at very high doses has not been shown to produce C-cell proliferation in monkeys. Data from long term studies, such as the 1860-Lira-DPP-4 trial, have not shown any increase in the mean calcitonin level, which is the marker of C cell hyperplasia and medullary thyroid carcinoma, in patients treated with liraglutide [33,59].

### Cardiovascular safety

The large ongoing outcome trial LEADER (Liraglutide Effects and Actions in Diabetes, Evaluation of Cardiovascular Results) will investigate the safety profile of liraglutide in approximately 9,000 patients with T2DM. It will include patients with a high risk cardiovascular profile in a global setting [60]. EXSCEL (Exenatide Study of Cardiovascular Event Lowering) is a similar large study planned to investigate the safety of exenatideQW preparations. EXSCEL is a double-blind randomized, placebo controlled, multi-national superiority trial in patients with T2DM. It aims to compare the impact of including exenatide as part of usual care versus usual care without exenatide on major cardiovascular outcomes. A total of 9,500 patients will be recruited and will be followed for a minimum of four years [61]. TECOS (Trial Evaluating Cardiovascular Outcomes with Sitalgliptin) hopes to investigate safety and cardiovascular outcomes with sitagliptin. TECOS is a double-blind randomized, placebo controlled, multi-national trial in patients with T2DM. TECOS aims to compare the impact of adding sitagliptin as part of usual care versus usual care without sitagliptin on cardiovascular outcomes. A total of 14,000 patients will be followed for a minimum of three years [61].

### Patient reported outcome measures and satisfaction

Diabetes mellitus, its treatment and its complications often affect a patient's quality of life [8]. Patient reported treatment outcomes may provide the data on health related quality of life as well information on patients' perceptions on efficacy, tolerability and preferences about a particular therapy. Higher patient satisfaction may indicate better compliance with the therapy [62-64].

In the 1860-Lira-DPP-4 study group open label trial, patients' treatment satisfaction was assessed using the Diabetes Treatment Satisfaction Questionnaire (DTSQ). The increase in patients' treatment satisfaction from baseline was significantly higher with 1.8 mg liraglutide than with sitagliptin (4.35 versus 2.96, *P *= 0.03), but the increase with 1.2 mg liraglutide versus sitagliptin was not significant. Patients reported significantly greater improvement in treatment satisfaction with liraglutide 1.8 mg than sitagliptin on three items: 'current treatment' (difference = 0.35; *P *= 0.01), 'recommend' (difference = 0.41; *P *= 0.003) and 'continue' (difference = 0.44; *P *= 0.01). Patients perceived themselves less hyperglycemic on either of the doses of liraglutide compared to sitagliptin (*P *<0.05). There was no difference between liraglutide and sitagliptin on DTSQ items relating to treatment convenience and flexibility, indicating that patients were no less satisfied with the injectable than with the oral agent [65].

There was no significant difference in all five domains of the IWQOL total score between exenatide once a weekly and sitagliptin (5.15 versus 4.56). A greater improvement in overall treatment satisfaction was recorded with exenatide than with sitagliptin (difference 1.61, *P *= 0.0406). However, the DURATION 2 was a double dummy trial with all of the patients receiving a tablet as well as an injection. Hence, it is more difficult to tease out the differences between therapies [35].

In the double blind placebo controlled DURATION 4 trial there was no significant difference in weight-related quality of life, binge-eating behavior or health status between exenatide QW and sitagliptin monotherapy.

## Conclusions

In the clinical trials, both types of incretin-based therapies are effective in improving hyperglycemia; however, as suggested by the proof of concept study, the magnitude of glycemic improvement was significantly higher with GLP-1R agonists and was consistent in the order of estimated mean treatment difference in HbA1c of 0.34% to 0.63% over and above that obtained with DPP-4 inhibitors. Greater HbA1c reduction with GLP-1agonists is probably due to pharmacological concentrations of free (non-albumin-bound) GLP-1 agonists [31,66]. DPP-4 inhibitors achieve two to three times increment in the native GLP concentration. However, several fold higher levels of GLP-1 agonist leads to greater stimulation of GLP-1 receptor [66]. Similarly, there is also a significantly greater weight loss (estimated mean treatment difference of -1.5 to -2.53 Kg) associated with GLP-1 agonists compared to DPP-4 inhibitors. This is most likely due to reduced calorie intake and central satiety effects. Although the differences in FPG were not evident during the initial short term proof of concept study, the longer term RCTs have consistently shown greater improvements in FPG with GLP-1 agonists as compared to DPP-4 inhibitors. Sitagliptin has a similar pharmacokinetic half-life to liraglutide (about 12 hours) but the increase in endogenous GLP-1 concentrations with DPP-4 inhibitors occurs mainly after meals. Thus, fasting concentrations of active GLP-1 remain fairly low overnight, so reductions in FPG concentrations with sitagliptin are low compared with liraglutide. While GLP-1 agonists are injected, DPP-4 inhibitors are taken orally and, although it is often stated that patients resist injectable therapies, published data suggest this is not by any means a universal finding. The results from the open label 1860 trial with liraglutide suggest patients were no less satisfied with injectable therapy compared to oral DPP-4 inhibitors and, in fact, were more satisfied in the 1.8 mg liraglutide arm compared to sitagliptin [65].

In general, the efficacy and safety of the incretin based agents from both classes have been shown to be durable. Their safety with longer term use will be ascertained by currently ongoing outcome trials (LEADER, EXSCEL, and TECOS) [60,61].

Similarly, although the currently marketed DPP-4 inhibitors appear to be comparable as a class regarding the degree of glycemic improvement, only sitagliptin was tested in these direct head to head comparisons. However, sitagliptin is the most widely prescribed DPP-4 inhibitor.

As with the other therapies, the selection of an incretin based agent for glycemic control in patients with T2DM should be individualized, taking into consideration the aims and intensity of glycemic improvement, tolerability of the therapy, the effect of such therapy on the various co-existing morbidities while assuring the therapy is acceptable and safe for patients in the longer term.

## Abbreviations

bid: twice a day; DPP-4 inhibitors: dipeptidylpeptidase-4 inhibitor; DTSQ: Diabetes Treatment Satisfaction Questionnaire; EMA: European Medicine Agency; EQ-5D: European Quality of Life - 5 Dimension; FDA: Food and Drug Administration; FPG: fasting plasma glucose; GIP: glucose-dependent insulinotropic peptide; GLP-1: glucagon like peptide 1; HbA1c: hemoglobin A1c; IGT: impaired glucose tolerance; PPG: postprandial glucose; RCT: randomized controlled trial; SMBG: self-monitoring blood glucose; SU: sulfonylureas; T2DM: type 2 diabetes mellitus;TZD: thiazolidinediones.

## Competing interests

KN and RK declare they have no competing interests. KK has acted as a consultant and speaker for Novartis, Novo Nordisk, Sanofi-Aventis, Lilly and Merck Sharp & Dohme. He has received grants in support of investigator and investigator initiated trials from Novartis, Novo Nordisk, Sanofi-Aventis, Lilly, Pfizer, Boehringer Ingelheim and Merck Sharp & Dohme. MJD has received funds for research, honoraria for speaking at meetings and has served on advisory boards for Lilly, Sanofi-Aventis, Merck Sharp & Dohme and Novo Nordisk.

## Authors' contributions

KN and RK drafted the manuscript. MD and KK critically reviewed the manuscript. All authors read and approved the final manuscript.

## Pre-publication history

The pre-publication history for this paper can be accessed here:

http://www.biomedcentral.com/1741-7015/10/152/prepub
